
JOURNAL INFORMATION
==============================
NLM Title Abbreviation: Innov Surg Sci
Journal ID: Innov Surg Sci
NLM Journal ID: 101708165
Journal ID: iss

Innovative Surgical Sciences

EISSN: 2364-7485
Publisher: De Gruyter

ARTICLE INFORMATION
==============================
PMCID: PMC10216888
DOI: 10.1515/iss-2023-9006
Article ID: iss-2023-9006
Article version: 1
Subjects: Article

Abstract DGG

Publication date: 2023 Mar
Electronic publication date: 2023 Mar 13
Volume: 8
Issue: Suppl1
First page: 129
Last page: 136
Copyright: © the Author(s), published by De Gruyter
Copyright year: 2023
Copyright holder: the Author(s), published by De Gruyter
License: This work is licensed under the Creative Commons Attribution 4.0 International License.
License URL: https://creativecommons.org/licenses/by/4.0/

Page count: 8

==============================

**Hybrid Approach for treatment of a giant left subclavian artery aneurysm involving the vertebral artery utilizing TEVAR and open supra aortic debranching. A case report**
ID: 305

**Kategorie: DGG - Neues aus der Gefäßchirurgie**

Peter Donndorf[1], Justus Groß[1], Peter Ouvrier[2], Clemens Schafmayer[1]

[1]*Universitätsmedizin Rostock, Klinik für Allgemein-, Viszeral-, Thorax-, Gefäß- und Transplantationschirurgie, Rostock, Deutschland*, [2]*Universitätsmedizin Rostock, Klinik für Anästhesiologie, Intensivmedizin und Schmerztherapie, Rostock, Deutschland*

**Background:**
A 63year old female was referred to our department with a pulsatile left supra clavicular tumor for further diagnostic and treatment. Medical history included Marfan syndrom, replacement of the aortic valve and the ascending aorta well as open thoracoabdominal aortic replacement.

**Materials and methods:**
Contrast enhanced Computed Tomography (CT) revealed a giant aneurysm of the proximal and middle left subclavian artery with a maximum diameter of 5.5cm. Due to the proximal extent of the aneurysm open surgical repair would have required a re-re sternotomy approach which was considered technically very high risk.
Likewise complete endovascular exclosure was deemed uncertain due to the large size and proximal extent of the aneurysm without an adequate proximal landing zone.
We opted therefore for an alternative indirect hybrid approach combining a thoracic aortic stent graft (TEVAR) covering the aortic origin left subclavian artery and left carotid-subclavian bypass as well as transposition of the left vertebral artery into the left common carotid artery for aneurysm exclusion.
First a 28x40mm Relay Pro aortic stent graft (Terumo Aortic, UK) was implanted in the distal aortic arch for proximal closure of the aneurysm. Next the left common carotid as well as the left vertebral artery were exposed via a left cervical incision and transposition of the left vertebral artery into the left common carotid artery was performed, facilitating save ligation of the proximal left vertebral artery arising from the aneurysm. Finally the distal left subclavian artery was exposed via a separate infraclavicular incision. A 8 mm Dacron prosthesis was anastomosed in an end-to-end fashion to the distal subclavian artery creating a carotid-subclavian bypass. The distal end of the mid left subclavian artery was ligated thereby completing aneurysm exclusion.

**Results:**
The patient had an uneventful postoperative course and was discharged at post-operative day six. A control CT prior hospital discharge showed sufficient exclosure of the subclavian aneurysm and unobstructed perfusion of the transposed left vertebral artery as well as the distal left subclavian artery.

Open Access. © 2023 The Author(s), published by De Gruyter. This work is licensed under the Creative Commons Attribution 4.0 International License.

**Conclusion:**

Large subclavian artery aneurysms are rare but can occur in certain patient subgroups, e.g. Marfan Syndrom. Due to their life-and limb threatening nature invasive treatment is indicated for large aneurysms. The hybrid treatment approach presented here was chosen due to multiple prior open surgical procedures creating a „hostile thorax“ making open access to the aneurysm technically very difficult. Our integrated concept combines well established endovascular (TEVAR) and open (supra-aortic debranching) procedures in order to achieve safe and reliable aneurysm exclosure. Although endovascular procedures should always be considered carefully when treating aneurysmal disease in Marfan patients, we believe that in cases like ours with a preventive setting and a high technical risk of complete open surgical repair the use of a routine endovascular procedure like TEVAR in combination with a tailored open surgical part for a less invasive and still efficient aneurysm treatment is justified. Our case is a good example how growing confidence of vascular surgeons in combining endovascular and open surgical procedures facilitates safe and efficient treatment even of rare and anatomically challenging vascular pathologies.

**female empowerment- Die Gefäßchirurginnen des Chirurginnen e.V.**

ID: 625

**Kategorie: DGG - Gefäßchirurgie ist weiblich**

Werra Ursula[1], Carola Wieker[2]

[1]*Uniklinik Köln, Gefäßchirurgie, Köln, Deutschland*, [2]*Universitätsklinikum Heidelberg, Heidelberg, Deutschland*

**Background:**

„Women in surgery- ending patriachy“titelte 2019 das journal of the royal society of medicine. “Women in medicine are fighting back.” lautet der erste Satz.

Aber gegen was wird hier gekämpft? Bellini et al. beschrieben einmal: DER Chirurg „is male, caucasian, busy, heroic, selected for perfection, sometimes grumpy when seeing sub-optimal performance in others, often inadvertently bullying”. [2]

2021 zählte die Bundesärztekammer 2300 berufstätige Gefäßchirurg*innen. 609 davon waren Frauen. Die Kolleg*innen in Weiterbildung werden hier nicht erfasst. In der Statistik werden 119884 chirurgisch tätige Kolleg*innen ohne Facharztbezeichung geführt, davon 69718 Frauen.

Also haben wir 57% Frauen insgesamt in der chirurgischen Weiterbildung, jedoch nur 19% Frauen im Bereich der niedergelassenen Gefäßchirurg:innen und 26% Frauen unter den stationär tätigen Fachärzt*innen. Und nur knapp jeder zehnte gefäßchirurgische Chef ist eine Chefin.

2018 evaluierte das American Journal of Surgery ob vielleicht die Abbruchraten der Weiterbildung eine Rolle spielten. Zwar bezog sich die Erhebung auf Allgemeinchirurgie, abgebrochen wurde jedoch über alle Geschlechter gleich mit ca. 21%.

**Materials and methods:**

2018 evaluierte das American Journal of Surgery ob vielleicht die Abbruchraten der Weiterbildung eine Rolle spielten. Zwar bezog sich die Erhebung auf Allgemeinchirurgie, abgebrochen wurde jedoch über alle Geschlechter gleich mit ca. 21%.

Woran liegt es dann? Was fehlt (jungen) Medizinerinnen und Chirurginnen in einem solchen Moment? Vorbilder. Vorbilder, die Frau bestärken, dass sie in der Chirurgie genau richtig ist; mit denen man die Möglichkeit hat über erlebte Verhaltensweisen zu diskutieren. Vorbilder, die bestärken den Chef/ die Chefin doch bezüglich einer Beförderung anzusprechen und helfen Strategien zu entwickeln- zum Beispiel wie der eigene Lebensentwurf oder Familie UND Karriere vereinbar werden.

**Results:**

In Deutschland gibt es seit dem 5.1. 2021 Die Chirurginnen e.V. Ein Verein von inzwischen über 1200 Chirurginnen aus dem deutschsprachigen Raum. Ca. 70 Gefäßchirurginnen sind bereits Mitglied. Neben dem jederzeit möglichen direkten Austausch über eine datensichere medizinische Plattform gibt es ein Mentoringprogramm oder Fortbildungsangebote.Es bestehen enge Kooperationen mit Projekten wie OPidS oder mit dem deutschen Ärztinnenbund. Der Verein ist auch in den sozialen Medien oder auf Kongressen präsent und gewann 2022 den Impact of Diversity Award in der Kategorie Gender Inclusion.

**Conclusion:**
Nur wenn die Chirurgie nicht mehr vorrangig als „männliche“ Domäne gilt, wird es auch gelingen, weiblichen Nachwuchs für operative Fächer zu gewinnen und zu halten. Ein großes Ziel des Vereins ist es, junge Frauen für die Chirurgie zu begeistern und sie zu ermutigen. Ob zum Schritt in die Selbstständigkeit ist oder zur Übernahme von Leitungsfunktionen in der Klinik und Habilitation- und nicht zuletzt zum berufspolitischen Engagement.
Wir möchten einen Überblick geben über die aktuelle Situation und einen Einblick in unsere Vereinsarbeit ermöglichen.

**Patency and Re-Intervention rates after Endovascular or Surgical Treatment of popliteal artery entrapment syndrome (PAES): a systematic review.**
ID: 803

**Kategorie: DGG - Neues aus der Gefäßchirurgie**

Abhay Setia[1], Sonal Kohli[1], Claus-Georg Schmedt[1]

[1]*Diakoneo Diak Klinikum Schwäbisch Hall, Schwäbisch Hall, Deutschland*

**Background:**
Popliteal artery entrapment syndrome (PAES) is an important differential diagnosis in young patients with intermittent claudication and without atherosclerotic risk factors. The aim of this systematic review was to put a light on the revascularization techniques to treat PAES and report the patency and re-intervention rates in this patients' group.

**Materials and methods:**
Multiple databases; Pubmed, Embase, Web of Science, Scopus and were searched for studies, case series, prospective trials, retrospective studies and randomized controlled trials. Case reports or series, prospective and retrospective studies reporting treatment of poplitea artery entrapment syndrome in any age-group reporting surgical treatment and atleast one follow-up after discharge were included. Book chapters, Books, Conference papers, Review articles and studies not providing any follow-up data, studies not available in English and studies with only abstracts were identified and excluded from the review. Studies reporting treatment of PAES with only Botulinum toxin injection were excluded.

**Results:**
A total of 41 studies matched the criteria. Age of patients ranged from 7-67 years. Symptoms ranged from claudication to rest pain and lesions. Duration of symptoms ranged from 1-2100 months. Typ 2 PAES was most commonly reported. Posterior approach was the preferred. Operative techniques were myotomy with or without revascularization, which was carried out with bypass or local endarterectomy and patch reconstruction of the artery. Follow-up range was 1-207 months. The 1-year, 3-Y and 5-Y patency rates ranged from 84.6-100%, 74.6-100% and 74.6-100% respectively. Secondary patency at 2-Y was 100%. Less than 20% patients require redo surgery within 5 years. All patients undergoing local TEA and patch reconstruction without myotomy failed during the follow-up period. Failed bypass resulted in amputation in a few.

**Conclusion:**

The patency rates are acceptable after vascular reconstructions for PAES. Local TEA and patch reconstruction was associated with high failure rates and should be avoided. Regular surveillance should be offered for patients undergoing vascular reconstructions for PAES.

**Association between hospital policy regarding type of treatment and outcomes of carotid endarterectomy. Secondary data analysis of the German statutory quality assurance database**
ID: 842

**Kategorie: DGG - Neues aus der Gefäßchirurgie**

Lan Zang[1], Felix Kirchhoff[1], Naher Shamsun[1], Stefan Saicic[1], Michael Salvermoser[1], Vanessa Lohe[1], Volker Schmidt[1], Eva Knipfer[1], Bianca Bohrmann[1], Christoph Knappich[1], Michael Kallmayer[1], Hans-Henning Eckstein[1], Andreas Kühnl[1]

[1]*Klinikum rechts der Isar der Technischen Universität München, München, Deutschland*

**Background:**
Since guidelines recommend carotid endarterectomy (CEA) as the first-line procedure for the majority of patients with an (a)symptomatic mid-to-high grade carotid stenosis, carotid artery stenting (CAS) is considered as an alternative under certain conditions. The indication for CEA or CAS should be determined by a multidisciplinary team. In Germany, only about 50% of hospitals providing both, CEA and CAS. In contrast, 40% and 10% of hospitals providing CEA only or CAS only, respectively. This study aimed to assess whether hospital policy regarding type of treatment is associated with the perioperative stroke or death rate.

**Materials and methods:**
This study is part of the ISAR-IQ project (Integration and Spatial Analysis of Regional, Site-specific, and patient-level factors for Improving Quality of treatment for carotid artery stenosis), which analysis data from the nationwide German statutory quality assurance carotid database. Research was funded by Germany's Federal Joint Committee Innovation Fund (GBA Innovationsfonds, 01VSF19016 ISAR-IQ). Hospitals were categorized according to their policy regarding type of treatment: both CEA and CAS in the same institution or CAS or CEA only). The primary outcome event was any perioperative stroke and death until discharge. Secondary outcomes were all strokes, all cause death and the combined rate of major stroke and death. Adjusted relative risks (RRs) were assessed by multivariable multilevel regression analysis.

**Results:**
A total of 201,330 patients with symptomatic and asymptomatic carotid stenosis undergoing CAS or CEA between 2012 and 2016 were included. The overall stroke and death rate was 2,1%. In centers providing both, CEA and CAS stroke and death rates were lower (CEA 2.0%, CAS 2.4%) compared to centers providing CEA only (2.1%) or CAS only (3.0%). The multivariable regression analysis showed significant higher perioperative stroke and death rate in the CEA only group (1.12 aOR, 1.03–1.21 95% CI) and CAS only group (1.26 aOR, 1.04–1.52 95% CI) versus the combined CEA and CAS group. Centers providing CAS only showed significant higher perioperative stroke rate

(1,25 aOR, 1.01–1.55 95% CI) and centers providing CEA only showed significant higher death rate (1,30 aOR, 1.12–1.50 95% CI) compared to centers providing both.

**Conclusion:**

A hospital policy of offering CEA and CAS in the same institution is associated with a lower risk of perioperative stroke and death than a policy of performing CEA or CAS only. These data support the recommendation that CEA or CAS should be performed only in centers that offer both in order to provide an individualized approach in carotid patients.
